# Pervasive glycative stress links metabolic imbalance and muscle atrophy in early-onset Parkinson's disease

**DOI:** 10.1016/j.molmet.2025.102163

**Published:** 2025-05-07

**Authors:** Natalia Prudente de Mello, Michelle Tamara Berger, Kim A. Lagerborg, Yingfei Yan, Jennifer Wettmarshausen, Susanne Keipert, Leopold Weidner, Janina Tokarz, Gabriele Möller, Stefano Ciciliot, Safal Walia, Yiming Cheng, Margarita Chudenkova, Anna Artati, Daniela Vogt Weisenhorn, Wolfgang Wurst, Jerzy Adamski, Roland Nilsson, Giovanni Cossu, Agnita Boon, Anneke Kievit, Wim Mandemakers, Vincenzo Bonifati, Mohit Jain, Martin Jastroch, Philippe Schmitt-Kopplin, Fabiana Perocchi, Kenneth Allen Dyar

**Affiliations:** 1 Institute for Diabetes and Obesity, Helmholtz Munich, Munich, Germany; 2 Graduate School of Systemic Neurosciences, Ludwig Maximilian University, Munich, Germany; 3 Analytical Food Chemistry, Technical University of Munich, Munich, Germany; 4 Environmental Health Center, Helmholtz Munich, Munich, Germany; 5 Departments of Medicine and Pharmacology, UC San Diego School of Medicine, La Jolla, CA, USA; 6 Department of Molecular Biosciences, The Wenner-Gren Institute, Stockholm University, Stockholm, Sweden; 7 Institute for Diabetes and Cancer, Helmholtz Munich, Munich, Germany; 8 German Center for Diabetes Research, Munich, Germany; 9 Department of Molecular Medicine, Pavia University, Pavia, Italy; 10 Venetian Institute of Molecular Medicine, Padova, Italy; 11 School of Medicine and Health, Technical University of Munich, Munich, Germany; 12 Metabolomics and Proteomics Core Facility, Helmholtz Munich, Munich, Germany; 13 Institute of Developmental Genetics, Helmholtz Munich, Munich, Germany; 14 Munich Cluster for Systems Neurology, Munich, Germany; 15 Department of Developmental Genetics, TUM School of Life Sciences, Technical University of Munich, Munich, Germany; 16 German Center for Neurodegenerative Diseases, Munich, Germany; 17 Institute of Experimental Genetics, Helmholtz Munich, Munich, Germany; 18 Institute of Biochemistry, Faculty of Medicine, University of Ljubljana, Ljubljana, Slovenia; 19 Department of Biochemistry, Yong Loo Lin School of Medicine, National University of Singapore, Singapore; 20 Department of Medicine, Karolinska Institute, Karolinska University Hospital, Stockholm, Sweden; 21 Department of Neuroscience, Brotzu Hospital, Cagliari, Italy; 22 Department of Neurology, Erasmus MC, University Medical Center Rotterdam, Rotterdam, the Netherlands; 23 Department of Clinical Genetics, Erasmus MC, University Medical Center Rotterdam, Rotterdam, the Netherlands; 24 Institute of Neuronal Cell Biology, Technical University of Munich, Munich, Germany

**Keywords:** Parkinson's disease, Muscle atrophy, Glycobiology, Glycative stress, Advanced glycation endproducts (AGEs), Biomarkers

## Abstract

**Objective:**

Parkinson’s disease (PD) is recognized as a systemic condition, with clinical features potentially modifiable by dietary intervention. Diets high in saturated fats and refined sugars significantly increase PD risk and exacerbate motor and non-motor symptoms, yet precise metabolic mechanisms are unclear. Our objective here was to investigate the interplay between diet and PD-associated phenotypes from a metabolic perspective.

**Methods:**

We explored PARK7 KO mice under chronic glycative stress induced by prolonged high-fat high-sucrose (HFHS) diet. We investigated metabolic consequences by combining classical metabolic phenotyping (body composition, glucose tolerance, indirect calorimetry, functional assays of isolated mitochondria) with metabolomics profiling of biospecimens from mice and PD patients.

**Results:**

We found this obesogenic diet drives loss of fat and muscle mass in early-onset PD mice, with a selective vulnerability of glycolytic myofibers. We show that PD mice and early-onset familial PD patients are under pervasive glycative stress with pathological accumulation of advanced glycation end products (AGEs), including N-α-glycerinylarginine (α-GR) and N-α-glycerinyllysine (α-GK), two previously unknown glycerinyl-AGE markers.

**Conclusions:**

Our results offer the first proof for a direct link between diet, accumulation of AGEs and genetics of PD. We also expand the repertoire of clinically-relevant glycative stress biomarkers to potentially define at-risk patients before neurological or metabolic symptoms arise, and/or to monitor disease onset, progression, and effects of interventions.

## Highlights

• High-fat high-sucrose diet exacerbates muscle atrophy in PARK7 KO mice, a model of early-onset parkinsonism.
• Atrophy of glycolytic 2B myofibers is associated with accumulation of advanced glycation endproducts (AGEs).
• Identification of 2 novel arginine and lysine amide-AGEs in tissues and biofluids of PARK7 KO mice.
• Novel amide-AGEs distinguish patient fibroblasts with PARK7 KO early-onset Parkinson's disease from healthy controls.

Despite being one of the most prevalent neurodegenerative diseases (NDDs) and movement disorders [1], we currently lack sufficient understanding of PD pathogenesis. Although clinically distinct, PD and metabolic disorders are thought to present overlapping pathophysiological mechanisms involving complex gene-environment interactions [2]. Diets high in saturated fats and refined sugars contribute to obesity and type 2 diabetes (T2D), and are potent and established risk factors for PD development and worsening of motor and non-motor symptoms [3,4]. These in turn are accompanied by unintentional weight loss, reduced fat and muscle mass, and dysregulated lipid and glucose metabolism [5,6]. Non-enzymatic glycation is considered a common trigger for many age-related and chronic diseases [7], including NDDs and T2D. This is due to accumulation of AGEs, cytotoxic compounds derived from spontaneous reactions between reducing sugars and proteins, lipids, or nucleic acids [8]. AGEs trigger oxidative stress, inflammation, muscle atrophy, cataract formation, and protein aggregates, including Lewy bodies [9]. While systemic AGEs mostly arise from a combination of glucose exposure, defective glycolysis, and insufficient detoxification, ∼30 % are directly from dietary sources [7].

**Figure 1:**
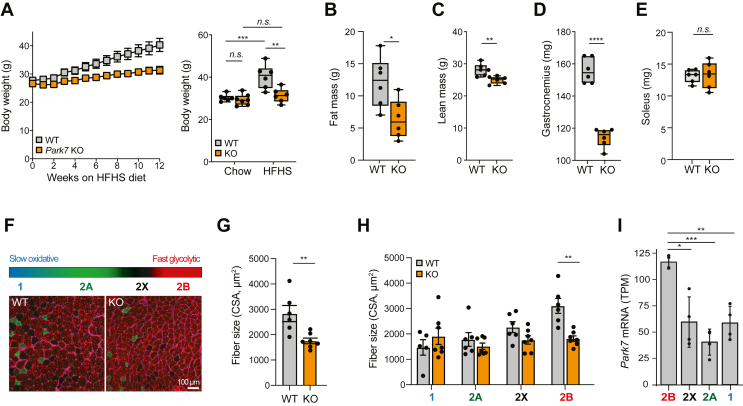
**HFHS diet drives resistance to body weight gain and muscle atrophy in early-onset PD.** (**A**) Body weight of WT and KO mice after 12 weeks of HFHS diet. (A–E) Box and whiskers plots showing quantification of fat (**B**), lean (**C**), gastrocnemius (**D**) and soleus (**E**) mass from WT and KO mice under HFHS diet. Whiskers show min to max, boxes represent lower and higher quartiles, and middle line is median (*n* = 6–7/group). (F) Transverse sections of gastrocnemius from WT and KO mice under HFHS diet stained with anti-myosin antibodies specific for type 1/slow (blue), fast type 2A (green) and fast type 2B (red) myosin heavy chains. Fast type 2X fibers are unstained and appear black. (G) Total fiber size in WT and KO muscles under HFHS diet (mean ± SEM; *n* = 6–7/group). (H) Fiber size according to fiber type (mean ± SEM; *n* = 6–7/group). (**I**) Normalized *Park7* mRNA expression in different mouse hindlimb myonuclei [15] (mean ± SEM; *n* = 4). *CSA*, cross sectional area; *TPM*, transcript per million. Data were analyzed using unpaired Student's *t*-test (**A**-**E**, **G, I**) or two-way ANOVA with Tukey's post hoc test (**H**); ∗∗∗∗*p* ≤ 0.0001, ∗∗∗*p* ≤ 0.001, ∗∗*p* ≤ 0.01, ∗*p* ≤ 0.05, *n.s.* not significant. (For interpretation of the references to colour in this figure legend, the reader is referred to the Web version of this article.)

To investigate potential diet-PD interactions we profiled a genetic model of early-onset PD [10], namely a mouse line lacking *Park7* (also called *Dj1*) under chronic glycative stress induced by prolonged high-fat high-sucrose (HFHS) diet [11]. *Park7* knockout mice (KO) failed to gain weight (Figure 1A) and had reduced fat (Figure 1B and Extended Data Figure 1a,b) and lean mass (Fig. 1C) compared to wild-type littermates (WT). While size and weight of internal organs were unaffected (Extended Data Fig. 1c-e), muscle mass of KO mice was greatly reduced (∼26 %) in glycolytic gastrocnemius (Fig. 1D) but not in oxidative soleus (Fig. 1E). In agreement with a recent report [12], we also noted 20 % atrophy of KO gastrocnemius muscles under chow diet, although total lean and fat mass were unchanged (Extended data Figure 1F–H). Impairment of muscle health, manifesting as wasting and fatigue, is often experienced by PD patients even prior to diagnosis [13]. We therefore sought to investigate potential underlying molecular mechanisms. Intriguingly, muscle atrophy in *Park7*-related PD mice occurred independently of any changes in systemic glucose tolerance, fasting glucose levels, food intake, locomotor activity, energy expenditure, and fuel selection (Extended Data Fig. 2) and, contrary to prior observations [14], was not due to any reprogramming of energy metabolism. Indeed, *Park7*-knockdown C2C12 myotubes showed no alterations in oxidative phosphorylation nor glycolytic activity (Extended Data Fig. 3). Protein abundance, assembly and function of respiratory chain complexes in KO gastrocnemius mitochondria seemingly remained unaltered, and levels of reactive oxygen species (ROS) were unaffected (Extended Data Fig. 4). Instead, gastrocnemius muscle fiber size analysis revealed a selective vulnerability of highly glycolytic type 2B compared to oxidative type 2X, 2A, or 1 myofibers (Figure 1F–H), consistent with *Park7*/PARK7 tissue- and -cell type-specific expression [15], [16] (Figure 1I and Extended Data Fig.5). Altogether, our findings recapitulate metabolic phenotypes associated with PD and suggest a link between glucose metabolism and muscle wasting.

**Figure 2:**
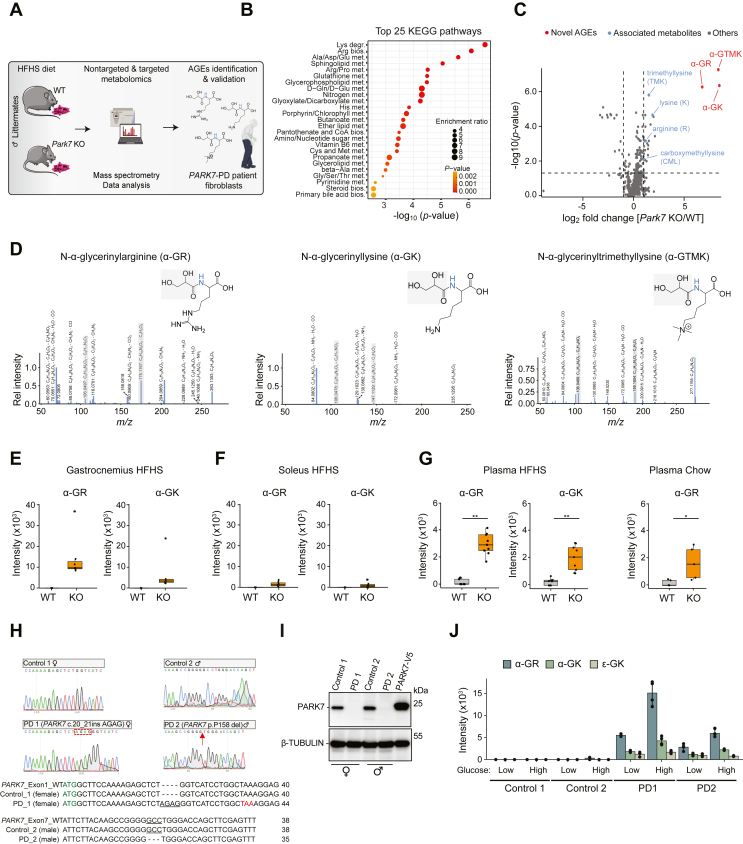
**Pervasive accumulation of AGEs in disease-relevant specimens from early-onset PD mice and patients.** (**A**) Scheme of metabolomics experiments. Figure elements created with SMART (Servier Medical Art, http://smart.servier.com/) and BioRender.com, and licensed under a Creative Common Attribution 4.0 International License. (**B**) KEGG pathway enrichment of significant RP-UHPLC-MS/MS metabolites in gastrocnemius muscles. (**C**) Volcano plot comparing HILIC-MS/MS quantified metabolites in gastrocnemius from WT and KO mice under HFHS diet. (**D**) Chemical structures of new glycerinyl-AGEs. (e–g) Box and whiskers plots showing quantification of glycerinyl-AGEs in gastrocnemius (**E**), soleus (**F**) and plasma (**G**) from WT and KO mice. Whiskers show min to max, boxes represent lower and higher quartiles, and middle line is median (*n* = 5–9/group). Data were analyzed using nonparametric Wilcoxon signed-rank test (**G**); ∗∗*p* ≤ 0.01, ∗*p* ≤ 0.05. (**H**) Sequence analysis of fibroblast DNA from two *PARK7-*related patients and healthy controls. The female (PD 1) and male (PD 2) patients carry a 4bp insertion in exon 1 (red box) leading to a premature stop codon (TAA) and a 3bp loss in exon 7 (red arrow), respectively. (**I**) Western blot analysis of PARK7 protein levels in healthy controls, patient fibroblasts, and V5-tagged PARK7-overexpressing HEK293 cells. (**J**) Glycerinyl-AGEs in fibroblasts from control and *PARK7*-related PD patients cultured under low (1.0 g/L) or high (4.5 g/L) glucose (mean ± SD; *n* = 4). (For interpretation of the references to colour in this figure legend, the reader is referred to the Web version of this article.)

To identify differences in systemic metabolism between healthy and early-onset PD conditions, we complemented metabolic phenotyping with mass spectrometry (MS)-based metabolomics in mouse tissues and early-onset familial PD patient fibroblasts (Figure 2A). First, we used reversed-phase ultrahigh-performance liquid chromatography-tandem mass spectrometry (RP-UHPLC-MS/MS) to explore metabolic signatures in WT and KO gastrocnemius muscles (Extended Data Table 1). In total, 97 of 358 mainly nonpolar metabolites were significantly altered in KO muscles, with 89 elevated and 8 reduced (Figure 2B and Extended Data Fig. 6), reflecting protein degradation and profound oxidative and glycative stress. While carnosine-related metabolites with antiglycating properties (e.g., N-acetylcarnosine and beta-alanine) were decreased, arginine and lysine derivatives were increased, including N-acetylarginine, trimethyllysine (TMK), fructosyllysine, a common Amadori product formed in early glycation, and N6-carboxymethyllysine (CML), a major AGE.

To broaden metabolome coverage and capture more polar metabolites we employed hydrophilic interaction liquid chromatography-tandem mass spectrometry (HILIC-MS/MS). We confirmed elevated arginine, lysine, CML, and TMK, in addition to a striking accumulation of three previously undescribed AGEs (Figure 2C). Their abundance was over 32-fold higher in KO gastrocnemius muscles compared to WT, and was exacerbated by HFHS diet (Extended Data Fig. 7a). Since we could not find any reference spectra for the new molecules in *Global Natural Product Social Molecular Networking* (GNPS) or MassBank Europe databases, we first predicted structures based on MS/MS fragmentation patterns (Figure 2D) and then verified them with commercially synthesized authentic standards and isotopically labeled gylcerinyl-amino acid model systems using d-glucose-^13^C_6_ (Extended Data Figure 7b,c). We unambiguously identified two molecules as N-α-glycerinylarginine (α-GR) and N-α-glycerinyllysine (α-GK), while the third molecule was predicted to be N-α-glycerinyltrimethyllysine (α-GTMK). Structures suggest these new AGEs originate from modification and subsequent degradation of N-terminal arginine and lysine residues and/or reaction with free amino acids to form ‘glycation free adducts’ [17]. In KO mice we detected ∼5-fold and ∼2-fold higher abundance of α-GR and α-GK, respectively, in glycolytic gastrocnemius muscles compared to oxidative soleus muscles (Figure 2E,F). Our data suggest relatively high rates of glycolysis in mouse glycolytic muscles may promote buildup of AGEs and contribute to muscle atrophy observed in *Park7*-related PD mice.

To evaluate the extent of AGE-related accumulation, we measured the new amide-AGEs in disease-relevant samples. AGE accumulation in plasma is considered detrimental due to chronic and sustained activation of AGE receptor (RAGE)-dependent signaling, promoting inflammation and oxidative stress [7]. We detected a steep accumulation of α-GR and α-GK in plasma from PD mice that was further increased by HFHS diet (Fig. 2G). Therefore, glycative stress may already be pervasive in early-onset forms of PD. We corroborated these findings in skin fibroblasts from two different early-onset familial PD patients, 28–29 years old at the time of disease onset, and each with a different mutation in *PARK7* (Fig. 2H). A female patient (PD 1) was homozygous for a 4bp insertion in exon 1 (c.20_21insAGAG), leading to frameshift and premature TAA stop codon, whereas a male patient (PD 2) was homozygous for a 3bp loss in exon 7 (p.P158del). Both mutations cause a complete loss of PARK7 protein compared to control fibroblasts from unrelated healthy donors (Figure 2I). Interestingly, PD 1 also reported cataracts, often due to excessive glycation of lens proteins [18]. Since we did not know what impact physiologically high levels of glucose [4.5 g/L] normally found in standard culture media would have on the formation of glycerinyl-AGEs, we cultured cells in the presence of normal fasting levels of glucose [1.0 g/L] or high glucose. As shown in Figure 2J, we detected selective accumulation of α-GR, α-GK, and the recently identified ε-GK [19] in patient fibroblasts but not in healthy controls. Interestingly, α-GR and α-GK levels were greater in the presence of higher glucose.

Altogether, our results are of broad biomedical relevance because they offer the first proof for a direct link between diet, accumulation of AGEs and genetics of PD. Indeed, while PARK7 mutations are rare, its function can be lost even in individuals without mutations, for example by oxidative stress and aging [20,21] and its expression is significantly reduced in lymphocytes of patients at risk for developing PD [22]. Furthermore, given the progressive accumulation of AGEs with aging, it is conceivable that glycation stress is also a risk factor for idiopathic PD. Indeed, preliminary studies suggest a correlation between high plasma levels of AGEs, glycated hemoglobin and PD [[23], [24], [25]]. With limited treatment options, promoting healthier diets could at least limit formation and accumulation of AGEs and thereby reduce disease risk and severity [4]. Importantly, by identifying two additional AGEs readily detectable in body fluids and patient skin biopsies we expand the repertoire of clinically-relevant glycative stress biomarkers to define at-risk patients before neurological or metabolic symptoms arise, or to monitor disease onset and progression.

## 1 Methods

### 1.1 Contact for reagent and resource sharing

Further information and requests for resources and reagents should be directed to and will be fulfilled by Kenneth Dyar (kenneth.dyar@helmholtz-munich.de) and Fabiana Perocchi (fabiana.perocchi@helmholtz-munich.de).

### 1.2 Ethics statements

Animal experiments complied with the European directive 2010/63/EU of the European Parliament and were approved by the local animal welfare authority in Germany (District government of upper Bavaria No.55.2-1-54-2532-94-2012.

The cell lines from the two patients carrying *PARK7* variants were obtained within studies approved by local medical ethical committees of the Brotzu Hospital, Cagliari, and the Erasmus MC Rotterdam, conform to the principles of the Declaration of Helsinki. The participating subjects provided written informed consent for the use of the material for research purposes.

### 1.3 Animals, diet, and sample collection

Wildtype and *Park7* knockout mice on a C57BL6/J background (*Park7*_XE726_GT) were bred and maintained at the animal facilities of Helmholtz Munich. *Park7* KO mice were previously generated by gene trap insertion as described [10]. All mice were housed at 23 °C under a 12hr light–dark regimen with free access to water and a standard chow diet (#1310, Altromin, Germany). Since female mice are known to be more resistant to diet-induced obesity, we only examined male mice for the preclinical experiments presented here. At ∼12 weeks of age mice were randomized, and continued eating the chow diet, or were switched to a high-fat high-sucrose diet (58 % kcal from fat and sucrose, Research diets, D12331) for another 12 weeks. At ∼6 months of age mice were euthanized by cervical dislocation ∼3–4 h after the start of the light phase (∼9am–10am). Tissues were immediately collected, snap frozen in liquid nitrogen, and stored at −80 °C until subsequent use.

### 1.4 Immunofluorescence

Cryosections of gastrocnemius muscles were stained with rabbit polyclonal antibody against laminin (L9595, Sigma-Aldrich, 1:100 dilution) and monoclonal anti-myosin heavy chain (MyHC) antibodies produced in the lab of Stefano Schiaffino [26] and distributed by the Developmental Studies Hybridoma Bank (DSHB, University of Iowa): BA-D5 (IgG2b, supernatant, 1:100 dilution) specific for MyHC-I, SC-71 (IgG1, supernatant, 1:100 dilution) specific for MyHC-2A and BF-F3 (IgM, purified antibody, 1:100 dilution) specific for MyHC-2B. Type 2X fibers are not recognized by these antibodies, and so appear black. Four different secondary antibodies (Jackson ImmunoResearch) were used to selectively bind to each primary antibody: goat anti-rabbit, conjugated with Alexa 647 fluorophore; goat anti-mouse IgG1, conjugated with DyLight488 fluorophore (to bind to SC-71); goat anti-mouse IgG2b, conjugated with DyLight405 fluorophore (to bind to BA-D5); goat anti-mouse IgM, conjugated with Alexa594 fluorophore (to bind to BF-F3). Muscle cryosections, 8 μm thick, were incubated with M.O.M. IgG blocking solution (Vector Laboratories) for 1 h at room temperature, then briefly rinsed once with PBS. A solution with all the primary antibodies in phosphate-buffered saline (PBS) containing 1 % of bovine serum albumin (BSA) was then prepared, and sections were incubated overnight at 4 °C. After 3 washes (5 min each) with PBS, sections were incubated for 30 min at 37 °C with a solution with the four different secondary antibodies, diluted in PBS containing 1 % of BSA and 5 % of goat serum. After 3 washes with PBS (5 min each) and a brief rinse in water, sections were mounted with a saturated solution of ethyl-vinyl alcohol in PBS with 30 % glycerol. A control incubation with no primary antibodies was performed, and also control incubations with each primary antibody and non-specific secondary antibodies to exclude any possible cross-reaction. Pictures were collected with an epifluorescence and stage-motorized Leica DM6 B microscope, equipped with a Leica DFC 7000T camera. Single-color images were merged to obtain a whole muscle reconstruction with Leica LASX software, and images were analyzed with MATLAB using SMASH application (https://pubmed.ncbi.nlm.nih.gov/25937889/).

### 1.5 Indirect calorimetry and metabolic phenotyping

Total body fat and lean tissue mass was quantified by nuclear magnetic resonance (EchoMRI). Food intake, locomotor activity, energy expenditure, and respiratory exchange ratio (RER) were all monitored on a TSE system (Bad Homburg, Germany).

### 1.6 Isolation of skeletal muscle mitochondria from mice

Skeletal muscle (SKM) mitochondria from WT and *Park7* KO male mice were isolated by differential centrifugation [27]. Hind limb skeletal muscles were collected from mice after cervical dislocation. Muscle was placed in ice cold isolation buffer 1 (IB1) at pH 7.4 at 4 °C (100 mM KCL, 50 mM Tris - HCl, 2 mM EGTA, 0.5 % essentially fatty acid free BSA) and minced in small pieces. Tissue was washed several times to remove blood. IB1 was decanted and replaced by IB2 pH 7.4 at 4 °C (100 mM KCL, 50 mM Tris - HCl, 2 mM EGTA, 5 mM MgCl_2_, 1 mM ATP, 245.7 U/100 ml Protease Type VIII, 0.5 % essentially fatty acid free BSA). After 3.5 min incubation stirring on ice, samples were homogenized with a motor driven Teflon pestle with 3 strokes at 500 rpm. Homogenate was twice centrifuged at 10,400 × *g* at 4 °C for 10 min and pellet was gently resuspended and filled up with isolation buffer 1. Samples were centrifuged at 4000 × *g* at 4 °C for 10 min. Mitochondrial pellet was gently resuspended in a small volume of isolation buffer.

### 1.7 Measurements of glycolytic rate, mitochondrial bioenergetics, and ROS in isolated mitochondria and C2C12 cells

sh-*Park7* C2C12 myotubes and pLKO cells were seeded at a density of 10,000 cells/well in a Seahorse XFe96 Cell Culture Microplate. On the day of the experiment, medium was removed, and cells were washed twice with 200 μL of the respective assay medium for the mito-stress test (Agilent Base Medium supplemented with 25 mM glucose, 2 mM l-glutamine and 1 mM pyruvate, pH 7.4) or glycolysis (Agilent Base Medium supplemented with 2 mM l-glutamine, pH 7.4). Next, 180 μL of the assay medium for mito-stress test or glycolysis were added to each well and the plate was incubated for 60 min in a non-CO_2_ incubator at 37 °C. For the mito-stress test, 10× port solution of the following compounds were injected to reach the specified final concentration: oligomycin (1.5 μM), carbonyl cyanide m-chlorophenyl hydrazone (CCCP; 1.5 μM), and antimycin A/rotenone (4 μM/2 μM). For the glycolysis assay, glucose (10 mM), oligomycin (1.5 μM), 2-DG (100 mM), and antimycin A/rotenone (4 μM/2 μM).

For isolated mitochondria from gastrocnemius muscles of WT and *Park7* KO mice, mitochondrial respiration was measured as rates of O2 consumption (OCR, pmol/min) with a XFe96 extracellular flux analyser (Seahorse Bioscience). Mitochondria were quantified by BCA protein assay (Pierce BCA Protein Assay kit, Thermo Fisher cat. no. 23225) and diluted in 10 mM pyruvate/2 mM malate or 25 uM palmitoyl-carnitine/2 mM malate in ice cold Mitochondria Assay Solution (MAS) pH 7.2 (70 mM sucrose, 220 mM mannitol, 5 mM Kh2PO4, 5 mM MgCl2, 2 mM Hepes, 1 mM EGTA - KOH and 0.2 % (w/v) fatty acid-free BSA). 10 ul containing 3 ug of diluted SKM mitochondria were attached on the 96 well XF plate (Seahorse Bioscience) by centrifugation at 2000 × *g* at 4 °C for 20 min. Wells were gently filled up with 170 ul MAS buffer supplemented with corresponding substrates and the plate was immediately loaded onto the instrument. 10× port solution of the following compounds were injected to reach the specified final concentration: adenosine diphosphate (ADP, 4 mM), oligomycin (1.5 μM), carbonyl cyanide m-chlorophenyl hydrazone (CCCP, 15 μM), and antimycin A/rotenone (4 μM/2 μM). Horseradish peroxidase (HPR)-based quantification of reactive oxygen species was performed as described in [28].

### 1.8 Blue native page analysis of respiratory chain complexes

Blue-Native (BN) PAGE analysis was performed as previously described [29]. Briefly, 10 μg of mitochondria per lane were diluted at least 1:100 in ice-cold milli-Q water with proteinase inhibitors (Sigma-Aldrich, 5056489001), the pellet was then collected at 20,000 × *g* for 10 min at 4 °C, resuspended in ice-cold 1x BN Sample Buffer (Thermo Fisher Scientific, BN2003) with 1 % (w/v) digitonin and incubated on ice for 15 min. Afterwards, the sample was centrifuged at 20,000 × *g* at 4 °C for 30 min and the supernatant was transferred into a new pre-chilled tube with 0.25 % G-250 (Thermo Fisher Scientific, BN2004). BN-PAGE was performed at 4 °C on Native PAGE Novex 3–12 % Bis-Tris Protein gels (Thermo Fisher Scientific, BN1001) according to manufacturer's instructions, followed by overnight wet blot transfer at 30 V and 4 °C onto a 0.2 μM pore size PVDF membrane (Amersham, GE10600021). Immunoblotting was performed according to the standard protocol.

Second dimension (2D) analysis was performed as described in [30] with some modifications. Following the above described first dimension (1D) BN-PAGE, sample lanes for 2D were excised, incubated in 1x SDS sample buffer (Bio-Rad, 1610747) for 10 min and boiled shortly, followed by incubation in hot 1x SDS sample buffer for 15 min. As control, one well was loaded with 5 μg of input mitochondria previously diluted in 5 μL of 2x LDS dye (Thermo Fisher Scientific, 84,788) with 2.75 mM β-mercaptoethanol (Thermo Fisher Scientific, 21,985-023) and RIPA buffer (total volume 10 μL) and boiled for 5 min at 95 °C. The 2D well of the pre-cast NuPAGE 4 %–12 % Bis-tris 2D well gel (Thermo Fisher Scientific, NP0326BOX) was washed and filled with 1x MOPS running buffer (Thermo Fisher Scientific, NP0001). Lanes were then fitted onto the 2D and overlaid with 1x SDS sample buffer, followed by electrophoresis according to manufacturer's instructions. The proteins were then transferred by wet blot transfer on 0.2 μM pore size PVDF membrane (Amersham, GE10600021) and immunoblotted using NDUFB8, SDHA, CORE2, COX1 and ATP5a according to the standard protocol.

### 1.9 Fibroblast cell culture

Fibroblasts derived from human skin biopsies from patients and controls were maintained in high glucose (4.5 g/L glucose, Gibco) DMEM medium supplemented with 10 % FBS (FBS Superior, Biochrom) and 100 units/mL of penicillin and 100 μg/mL streptomycin (Gibco) at 37 °C and 5 % CO_2_ in a humidified atmosphere. Cells were frequently monitored to be free of mycoplasma contamination using the MycoAlert™ mycoplasma detection kit (Lonza) and the MycoAlert™ assay control set (Lonza).

For expression and mutation verification analyses, cells were harvested by trypsinization, washed once in warm PBS, and counted by using the Cellometer Auto T4 Plus (PeqLab). Cells were pelleted into aliquots containing 1 × 10^6^ cells and stored at −80 °C until further use.

For glycerinyl-AGEs determination by LC-MS/MS, 7 × 10^5^ cells were seeded into culture dishes (diameter 10 cm) with 12 mL of DMEM (4.5 g/L “High Glucose” or 1.0 g/L “Low Glucose”, Gibco) supplemented with FBS and penicillin/streptomycin as described above. Cells were grown for 3–5 days to reach confluence of ∼80 %. For harvesting, cells were washed twice with 13 mL of warm PBS before their metabolism was quenched by adding 3 mL of ice-cold extraction solvent (80/20 (v/v) methanol/water). Cells were then scraped off the culture dishes using rubber tipped cell scrapers (Sarstedt) and together with extraction solvent collected in 7 mL PeqLab tubes (cells and solvent of two 10 cm dishes were pooled in one tube) and stored at −80 °C until further use.

### 1.10 DNA isolation and verification of PARK7 mutations in fibroblasts

Genomic DNA was isolated from fibroblasts using the DNeasy Blood & Tissue Kit (Qiagen) according to the manufacturer's instructions. To verify the *PARK7* insertion mutation of patient 1, exon 1 of *PARK7* was PCR amplified using the intron/exon overlapping primers hPARK7_Ex1_for: 5′–TTTTTAAGGCTTGTAAACATATAAC-3′ and hPARK7_Ex1_rev: 5′–GACTTACCCCAGCTCGC-3′. The PCR products of around 130 bp were cloned into the pCRII-TOPO vector according to the manufacturer's instructions. Inserted sequences were verified by Sanger sequencing using standard M13 forward and reverse vector primers. To verify the *PARK7* deletion mutation of Patient 2, exon 7 of *PARK7* was PCR amplified from genomic DNA using the intronic primers hPARK7_Ex7_for1: 5′-CTGAAGGAGCAAGGAACTGGA-3′ and hPARK7_Ex7_rev1: 5′-GGAATGCTGGGTGCTATTACCT-3′ described by [31]. The resulting PCR products of around 1860 bp were purified by PCR Purification Kit (Macherey Nagel) and Sanger sequenced using the nested primers hPARK7_Ex7_for2: 5′-GCCCATTAGGATGTCACCTTT-3′ and hPARK7_Ex7_rev2: 5′-GCAGTTCGCTGCTCTAGTCTT-3′. Sequences were analyzed by the software SnapGene and sequence alignment performed using Clustal Omega (https://www.ebi.ac.uk/Tools/msa/clustalo/).

### 1.11 Protein expression of PARK7 in human cells

We transfected HEK293 cells with pLX304-hPARK7 plasmid. Aliquots of 1 × 10^6^ fibroblasts were suspended in 100 μL lysis buffer (20 mM Tris-HCL, 150 mM NaCl, 1 mM EDTA, 1 mM EGTA, 1 % Triton X-100, pH 7.5) supplemented with phosphatase inhibitor PhosSTOP and protease inhibitor C0mplete (Roche) and lysed by sonication. Total protein content was determined by Bradford assay (Bio-Rad) using BSA as reference protein. 20 μg of fibroblast lysate or 5 μg of HEK293 lysate were subjected to SDS-PAGE on a precast 4–15 % TGX Tris-Glycine Stain-Free Gel (Bio-Rad). Separated proteins were transferred within 30 min at constant voltage of 20 V to an Immobilon FL PVDF membrane (Millipore) using the Semi-Dry Transblot instrument (Bio-Rad). After blocking the membrane in 5 % milk powder in TBS, the blot was simultaneously incubated for 2 h at RT with the primary monoclonal antibodies rabbit-anti-DJ-1 (D29E5) XP® (Cell Signaling #5933) and mouse-anti-beta-tubulin TUB2.1 (Sigma #T5201), both at a dilution of 1:1000 in 2.5 % milk powder in TBS-T (0.1 % Tween-20). The blot was incubated overnight at 4 °C with the secondary antibodies goat anti rabbit-HRP (Sigma #A6154) and goat anti mouse-HRP (Dianova #115-035-068) at a dilution of 1:20,000 each in 2.5 % milk powder in TBS-T (0.1 % Tween-20). ECL signals were generated using the Pierce ECL 2 Western Blotting Substrate (Pierce) according to the manufacturer's instructions and detected using a Fusion FX6 Edge (Vilber Lourmat).

### 1.12 Reversed-phase ultrahigh-performance liquid chromatography (RP-UHPLC)-tandem mass spectrometry

Gastrocnemius muscle samples were randomized and homogenized with 1.4 mm ceramic beads in water (15 μL/mg) at 4 °C as described in [32]. To extract metabolites and to precipitate the protein, 500 μL methanol extraction solvent containing recovery standard compounds was added to each 100 μL of tissue homogenate. Supernatants were aliquoted and dried under nitrogen stream (TurboVap 96, Zymark, Hopkington, MA, USA) and stored at −80 °C until the UPLC-MS/MS measurements were performed. Two (i.e., early and late eluting compounds) aliquots were dedicated for analysis by UPLC-MS/MS in electrospray positive ionization and one for analysis by UPLC-MS/MS in negative ionization. Three types of quality control samples were included in each plate: samples generated from a pool of human ethylenediaminetetraacetic acid (EDTA) plasma, pooled sample generated from a small portion of each experimental sample served as technical replicate throughout the data set, and extracted water samples served as process blanks. Prior to UPLC-MS/MS analysis, the dried samples were reconstituted in acidic or basic LC-MS-compatible solvents, each of which contained eight or more isotopically labeled standard compounds at fixed concentrations to ensure injection and chromatographic consistency. For the UPLC-MS/MS platform we utilized a Waters (Milford, MA, USA) Acquity UPLC with Waters UPLC BEH C18–2.1 mm × 100 mm, 1.7 μm columns, a Thermo Scientific (Waltham, MA, USA) Q Exactive high resolution/accurate mass spectrometer interfaced with a heated electrospray ionization (HESI-II) source, and N Orbitrap mass analyzer operated at 35,000 mass resolution. One aliquot of the extracts reconstituted in acidic positive ion conditions, chromatographically optimized for more hydrophilic compounds (for early eluting compounds). In this method, the extracts were gradient eluted from the C18 column using water and methanol containing 0.05 % perfluoropentanoic acid (PFPA) and 0.1 % formic acid (FA). Another aliquot that was also analyzed using acidic positive ion conditions, but was chromatographically optimized for more hydrophobic compounds (for later eluting compounds), was gradient eluted from the same C18 column using methanol, acetonitrile, and water; containing 0.05 % PFPA and 0.01 % FA and was operated at an overall higher organic content. The basic negative ion condition extracts were gradient eluted from a separate C18 column using water and methanol containing 6.5 mM ammonium bicarbonate at pH 8. The MS analysis alternated between MS and data dependent MS2 scans using dynamic exclusion and a scan range of 80–1000 *m*/*z*. Metabolites were identified by automated comparison of the ion features in the experimental samples to a reference library of chemical standard entries that included retention time, molecular weight (*m*/*z*), preferred adducts, and in-source fragments as well as associated MS spectra and curation by visual inspection for quality control using proprietary software developed by Metabolon Inc. Only fully annotated metabolites were included for further evaluation. Data were normalized according to raw area counts, and then each metabolite scaled by setting the median equal to 1. Features with >40 % missing values were removed, and remaining missing values (7 %) were imputed with k-nearest neighbors algorithm. Biochemicals labelled with an asterisk (∗) indicate compounds that have not been officially confirmed with a standard, but we are confident in its identity.

### 1.13 Hydrophilic interaction liquid chromatography (HILIC)-tandem mass spectrometry

Mouse gastrocnemius muscle samples under HFHS or chow diet were analyzed. A total of 200 mg of 1 mm zirconium beads was added to a 2.0 mL impact resistant tube and placed on dry ice to pre-chill the tube. Tissue samples were then weighed, placed into the tube, and 1.0 mL of cold 80:20 methanol (Honeywell-Burdick & Jackson LC230-4):water (Fisher Chemical W6-4) was added. Muscle samples were homogenized by two rounds of three 15 s homogenization cycles at 6,400Hz in a Precellys 24 tissue homogenizer (Bertin Corp), with a 1-min cooling step on dry ice in between cycles. After homogenization, samples were placed in the −20 °C freezer for 30 min to allow for precipitation of protein. Samples were vortexed, transferred to a 1.5 mL Eppendorf tube, and centrifuged at 14,000rpm at 4 °C for 10 min. The supernatant was transferred to a clean tube and immediately dried in vacuo using a Thermo Savant vacuum concentrator operated at 35 °C. Once dry, samples were resuspended in a normalized volume of 80:20 methanol:water to result in a final concentration of 55 μg/μL. Tissue samples were transferred to LC-MS vials containing a 200 μl glass inserts were kept at 4 °C in the autosampler compartment until they were injected for analysis.

For muscle samples, 2 μl were injected on a Thermo QExactive orbitrap mass spectrometer coupled to a Thermo Vanquish UPLC system. Samples were randomized to alternate wildtype and knockout conditions. To analyze polar molecules, chromatographic separation was achieved using a Millipore (Sequant) Zic-pHILIC 2.1 × 150mm 5um column maintained at 25 °C with a flow rate of 0.3 mL/min. A 19-minute linear gradient starting from 90:10 acetonitrile (Honeywell-Burdick & Jackson LC015-4):20 mM ammonium bicarbonate (Fluka 1066-33-7) to 45:55 acetonitrile:20 mM ammonium bicarbonate was used to elute compounds. The Thermo Q-Exactive orbitrap mass spectrometer was operated in positive ion mode using a heated electrospray ionization (HESI) source at 35,000 resolution, 100 ms ion trap time for MS1 and 17,500 resolution, 50 ms ion trap time for MS2 collection. Data were collected over a mass range of *m*/*z* 67–1000. Auxiliary gas flow was used at a rate of 20 units, sheath gas flow rate of 40 units, sweep gas flow rate of 2 units, spray voltage of 3.5 kV, capillary inlet temperature of 275 °C, auxiliary gas heater temperature of 350 °C and an S-lens RF level of 45. All MS1 ions were isolated using a 1.0 *m*/*z* window for MS2 collection and fragmented using a normalized collision energy of 35. These fragmented ions were placed on dynamic exclusion for 30 s before being allowed to be fragmented again. For analysis, we imported collected data into the mzMine 2.20 software suite. Pure standards were used for identification of metabolites through manual inspection of spectral peaks and matching of retention time and MS1 accurate mass, with confirmation of identification through comparison to MS/MS fragmentation patterns.

### 1.14 Liquid chromatography-tandem mass spectrometry (LC-MS/MS) for structure validation of glycerinyl-AGEs

#### 1.14.1 Materials

d-(+)-Glucose (≥99.5 %), d-glucose-^13^C_6_ (≥99 %), l-arginine (≥98 %), and l-lysine (≥98 %) were purchased from Sigma-Aldrich (Steinheim, Germany). N-α-glycerinylarginine, N-α-glycerinyllysine, and N-ε-glycerinyllysine were purchased from Iris Biotech GmbH (Marktredwitz, Germany). Milli-Q purified water was from a Milli-Q Integral Water Purification System (18.2 MΩ, Millipore, Germany). Acetonitrile, methanol, and 2-propanol (all hypergrade for LC-MS) were purchased from Merck (Darmstadt, Germany). Formic acid (98 %, for mass spectrometry) was obtained from Honeywell Fluka (North Carolina, USA). Ammonium hydrogen carbonate (LC-MS grade) and ammonium formate solution 10 M in water (BioUltra grade) were purchased from Sigma-Aldrich (Steinheim, Germany). The API-TOF Reference Mass Solution Kit and ESI-L Low Concentration Tuning Mix were purchased from Agilent Technologies (Waldbronn, Germany). Even though we detected analogous fragmentation behaviors for α-GTMK, corresponding standards were not acquired due to challenges in synthesis. Other recently identified glycerinyl-modified amino acids [19] like asparagine (α-GN) and glutamine (α-GQ) were not further verified, as they did not show any significant changes in intensity in KO muscles, and hence, appeared to lack potential as atrophy-related biomarkers.

#### 1.14.2 Model system preparation

For reference ^13^C_3_H_4_O_3_-modified amino acids, model systems were prepared from amino acids, namely arginine and lysine, and d-glucose-^13^C_6_. Aqueous stock solutions (0.2 M) of d-glucose-^13^C_6_ and each amino acid were mixed 1:1 (*v*/*v*), respectively. d-Glucose-^13^C_6_ and amino acid standard solutions in Milli-Q purified water (0.1 M) were prepared as control samples. Samples were heated in closed glass vials for 2 h at 100 °C according to the protocol recently described [33]. Glycation products without isotopic tags were prepared analogously using regular d-(+)-glucose.

#### 1.14.3 Sample preparation

Samples were prepared in randomized order. Muscles and fibroblasts were handled on dry ice, plasma samples were handled on wet ice during all procedures. Muscles were weighed in pre-cooled NucleoSpin Bead Tubes (Macherey-Nagel, Düren, Germany) and homogenized in ice-cold 70:30 methanol:water (15 μL/mg tissue) after addition of 10 ceramic beads (diameter 1.4 mm) and 5 steel beads (diameter 2.4 mm) at 7,200 rpm for 8 cycles of 30 s followed by a 10 s pause between cycles using a Precellys Evolution Homogenizer (Bertin Corp., Rockville, Maryland, USA). Homogenates were transferred to empty extraction tubes and washed with 100 μL 70:30 methanol:water. Protein was precipitated from the homogenates by addition of pure methanol to reach 75:25 methanol:water. Homogenates were cleared by centrifugation at 13,000 rpm at 4 °C for 15 min. The supernatant was evaporated to dryness and resuspended in 80:20 acetonitrile:water (2 μL/mg tissue). After centrifugation at 13,000 rpm and 4 °C for 15 min, the supernatant was diluted 1:10 (*v/v*) in 80:20 acetonitrile:water for mass spectrometric analysis. For quality control, a pooled mixture of all reconstituted samples was prepared.

Plasma samples were thawed on wet ice and vortexed. For protein precipitation, 180 μL of ice-cold methanol was added to 20 μL of plasma. The mixtures were then vortexed and stored at −20 °C for 30 min. After centrifugation at 13,000 rpm and 4 °C for 15 min, 180 μL supernatant was evaporated to dryness at 30 °C and resuspended in 40 μL 50:50 acetonitrile:water. The reconstituted samples were centrifuged at 13,000 rpm and 4 °C for 15 min to remove insoluble materials, and the supernatants were transferred to vials for LC-MS/MS analysis. In addition, an equal amount of supernatant from each sample was mixed as a pooled quality control sample.

For fibroblast preparation, 960 mg of glass beads (diameter 0.5 mm, PEQLAB, Erlangen, Germany) and 2 g of ceramic beads (diameter 1.4 mm, PEQLAB, Erlangen, Germany) were added to each sample. Cell suspensions were homogenized at 5,500 rpm for 8 cycles of 25 s followed by a 10 s pause between cycles using a Precellys Evolution Homogenizer (Bertin Corp., Rockville, Maryland, USA). To normalize metabolomics data, 350 μL were taken from the homogenates for DNA level determination [34]. The remaining volume was cleared by centrifugation at 3,700 rpm and 4 °C for 15 min. Supernatants were evaporated to dryness at 30 °C and resuspended in Milli-Q purified water at a concentration factor of 1:50 (*v/v*). After centrifugation at 13,000 rpm and 4 °C for 15 min, the supernatant was transferred to vials for LC-MS/MS analysis. A pooled quality control sample for fibroblasts was prepared as described above.

#### 1.14.4 LC-MS/MS analysis for model systems

Prior to measurement, model systems were diluted 1:10 (*v*/*v*) in 80:20 acetonitrile:water. Samples were analyzed by a UHPLC (1290 Infinity II, Agilent, Waldbronn, Germany) coupled to a drift tube ion mobility (DTIMS) quadrupole time-of-flight (QToF) mass spectrometer (6560B, Agilent, Waldbronn, Germany). For hydrophilic interaction liquid chromatography (HILIC), a ZIC-cHILIC column (100 × 2.1 mm, 3 μm, 100 Å, zwitterionic, Merck, Darmstadt, Germany) was used. Samples were injected *via* partial-loop injection (10 μL). The flow rate was set to 0.5 mL/min and the column temperature was maintained at 40 °C. HILIC separation was run in gradient mode. Initial conditions were set to 0.01 % eluent A (acetonitrile:water, 5:95 (*v*/*v*), 5 mM ammonium formate, 0.1 % formic acid) and 99.9 % eluent B (acetonitrile:water, 95:5 (*v*/*v*), 5 mM ammonium formate, 0.1 % formic acid). After 2 min, eluent B was decreased to 56 % over the next 11 min. Eluent B reached 30 % within 1 min and finally 10 % after additional 0.1 min. This composition was maintained to the end of the run. The gradient was completed after 18.1 min. The MS was operated in positive electrospray ionization (ESI) mode within a mass range of 50–1700 *m*/*z* using QToF-only mode. Internal mass calibration and system tuning were performed using an ESI-L Low Concentration Tuning Mix solution prior to analysis and each MS spectrum was automatically recalibrated by continuously delivered API-TOF Reference Mass solution (purine *m*/*z* 121.0509 and HP-921 at *m*/*z* 922.0098) during analysis. The settings of the ion source were: capillary voltage 4000 V, nozzle voltage 10 V, dry gas temperature 250 °C, dry gas flow 12 L/min, nebulizer pressure 40 psi, sheath gas temperature 250 °C, sheath gas flow rate 11 L/min (both gasses nitrogen). Mass spectra were acquired with a scan rate of 8 Hz in data-dependent mode and the three highest MS [1] ions per precursor scan were fragmented using a normalized collision energy of 20 eV. Raw data were post-processed using Genedata Expressionist Refiner MS 15.0.7 (Genedata GmbH, Basel, Switzerland) applying chemical noise subtraction, intensity cutoff filter, chromatographic peak picking, isotope clustering, and retention time alignment. Further processing was performed in R software (version 4.2.0).

#### 1.14.5 LC-MS/MS analysis for muscle, plasma and fibroblast samples

Samples were analyzed in a randomized order using an Acquity UHPLC (Waters, Milford, MA, USA) coupled to a maXis QToF mass spectrometer (Bruker Daltonics, Bremen, Germany). HILIC analysis was performed on an Atlantis Premier BEH Z-HILIC column (150 × 2.1 mm, 1.7 μm, Waters, Milford, MA, USA). Flow rate was set to 0.5 mL/min. Column temperature was maintained at 40 °C. Separation was run in gradient mode. After 2.5 min of pre-equilibration time, 5 μL of sample were injected in partial-loop mode. Initial eluent conditions were set to 90 % eluent A (acetonitrile) and 10 % eluent B (20 mM ammonium hydrogen carbonate in Milli-Q purified water). After 2.0 min, eluent A was decreased to 45 % over the next 19.0 min and held at that composition for 3.5 min. Initial eluent conditions were restored over the next 0.1 min. The gradient was completed after 24.6 min. MS data was acquired in ESI(+)-mode within a mass range of 50–1500 *m/z* and a scan rate of 5 Hz in data-dependent mode. The three most abundant MS [1] ions per precursor scan were fragmented using a fixed collision energy of 25 eV. For structure validation, N-α-glycerinylarginine, N-α-glycerinyllysine, and N-ε-glycerinyllysine were additionally fragmented via multiple reaction monitoring (MRM) using an isolation window of 1 Da and a collision energy of 25 eV. External mass calibration was performed using an ESI-L Low Concentration Tuning Mix solution prior to each analysis. Ion source parameters were set to the following conditions: end plate offset 500 V, capillary voltage 4,500 V, nebulizer pressure 2.0 bar, dry gas flow 10 L/min at 200 °C (both gasses nitrogen). Data was post-processed as described above for model systems. Further data analysis was performed in R software (version 4.2.0).

#### 1.14.6 Consensus spectra computation

For model system data, consensus MS/MS spectra were computed with the R package MSnbase using the consensusSpectrum() function [35]. We retained mass peaks present in a minimal proportion of 5 % of spectra in the final consensus spectra. For peak aggregation, a maximum *m*/*z* merge distance of 0.005 Da was applied, and intensities of merged peaks were summed.

### 1.15 Statistical analyses

Sample size for all mouse experiments was 5–9 per genotype. When appropriate, data were analyzed by two-tailed unpaired Student's t-test or two-way ANOVA, followed by Tukey's or Sídák's multiple comparisons test using GraphPad Prism version 9. To statistically assess differences in metabolite intensities among experimental conditions, t-tests (unpaired, two-sided) or the nonparametric Wilcoxon signed-rank test were performed using the t.test() function from the stats R package. Data was subjected to log transformation and missing value imputation, if any, with a random number between minimum values across samples.

### 1.16 MetaboAnalyst

Enrichment analysis of altered metabolites was performed using MetaboAnalyst 5.0 [36] with default parameters and Kyoto Encyclopedia of Genes and Genomes (KEGG) IDs. Quantitative Enrichment Analysis was performed via the Enrichment Analysis module using the pathway based KEGG metabolite set library (84 metabolite sets based on human metabolic pathways).

## CRediT authorship contribution statement

**Natalia Prudente de Mello:** Writing – review & editing, Writing – original draft, Visualization, Investigation, Formal analysis, Data curation. **Michelle Tamara Berger:** Writing – review & editing, Validation, Formal analysis, Data curation. **Kim A. Lagerborg:** Formal analysis, Data curation. **Yingfei Yan:** Writing – review & editing, Visualization, Validation, Formal analysis. **Jennifer Wettmarshausen:** Validation, Investigation. **Susanne Keipert:** Investigation, Formal analysis, Data curation. **Leopold Weidner:** Writing – review & editing, Validation, Formal analysis, Data curation. **Janina Tokarz:** Writing – review & editing, Visualization, Methodology, Investigation, Formal analysis, Data curation. **Gabriele Möller:** Writing – review & editing, Visualization, Methodology, Investigation, Formal analysis. **Stefano Ciciliot:** Visualization, Validation, Investigation, Formal analysis. **Safal Walia:** Formal analysis. **Yiming Cheng:** Visualization, Formal analysis. **Margarita Chudenkova:** Validation, Investigation. **Anna Artati:** Methodology, Formal analysis. **Daniela Vogt Weisenhorn:** Writing – review & editing, Resources. **Wolfgang Wurst:** Resources, Funding acquisition. **Jerzy Adamski:** Resources, Methodology. **Roland Nilsson:** Writing – review & editing. **Giovanni Cossu:** Resources. **Agnita Boon:** Resources. **Anneke Kievit:** Writing – review & editing, Resources. **Wim Mandemakers:** Resources. **Vincenzo Bonifati:** Writing – review & editing, Resources, Funding acquisition. **Mohit Jain:** Supervision, Methodology. **Martin Jastroch:** Supervision, Resources, Methodology. **Philippe Schmitt-Kopplin:** Supervision, Resources, Project administration, Methodology. **Fabiana Perocchi:** Writing – review & editing, Writing – original draft, Supervision, Resources, Project administration, Methodology, Funding acquisition, Conceptualization. **Kenneth Allen Dyar:** Writing – review & editing, Writing – original draft, Visualization, Supervision, Resources, Project administration, Methodology, Funding acquisition, Formal analysis, Data curation, Conceptualization.

## Declaration of competing interest

The authors declare no competing interests.

## Data Availability

Data will be made available on request. Raw and processed metabolomics data are available at Mendeley Data (https://data.mendeley.com/datasets/x5rkxk7p57/1)
