# Supplementary material for: Pervasive glycative stress links metabolic imbalance and muscle atrophy in early-onset Parkinson's disease
Source: Mol Metab. 2025 May 7;97:102163. doi: 10.1016/j.molmet.2025.102163 (PMC12148662; doi:10.1016/j.molmet.2025.102163)
Supplement: Multimedia component 2 [file mmc2.pdf]

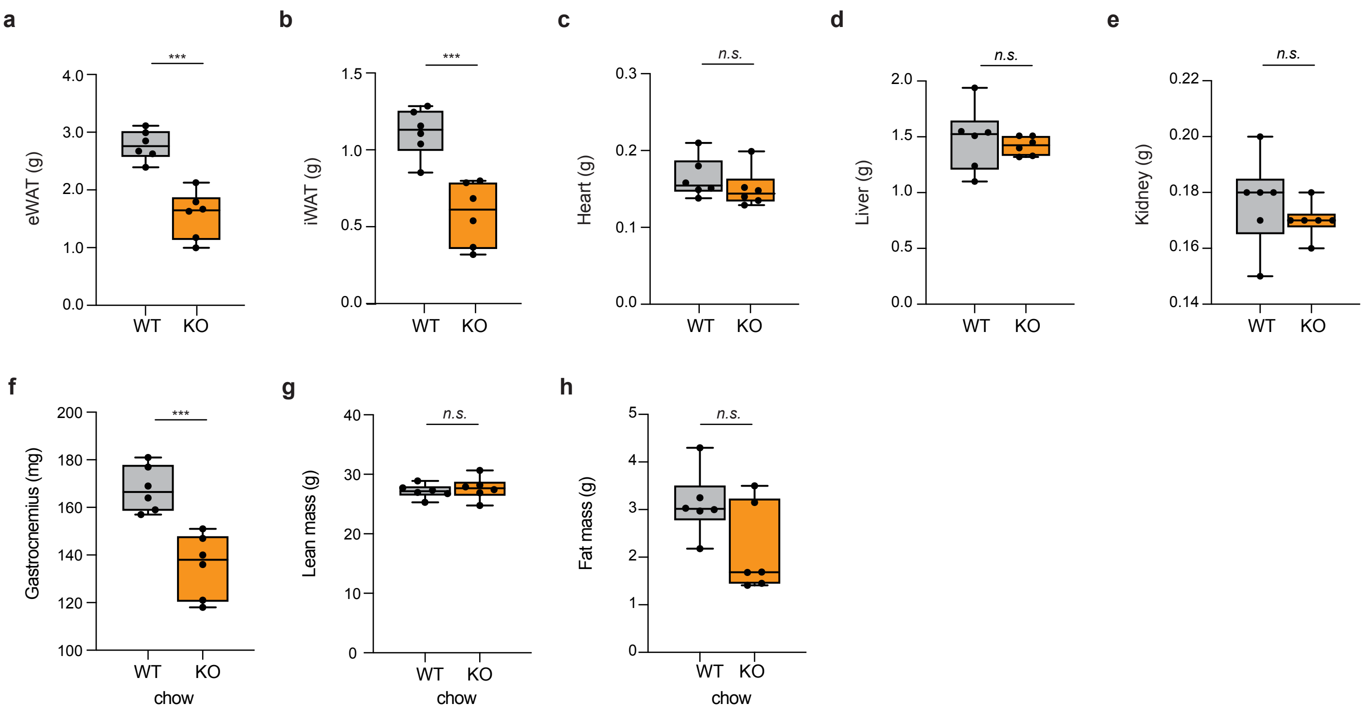

Extended Data Fig. 1: Loss of PARK7 leads to a decrease of fat mass during HFHS diet. Quantification of epididymal (**a**) and inguinal (**b**) white adipose tissues (WAT), heart (**c**), liver (**d**), and the kidneys (**e**) from WT and *Park7* KO mice on HFHS diet, as well as gastrocnemius (**f**), lean mass (**g**) and fat mass (**h**) on chow diet. Whiskers show min to max, boxes represent lower and higher quartiles, and middle line as median (n = 6) and were analyzed using unpaired t-test. ***$p \leq 0.001$, *n.s.* not significant.

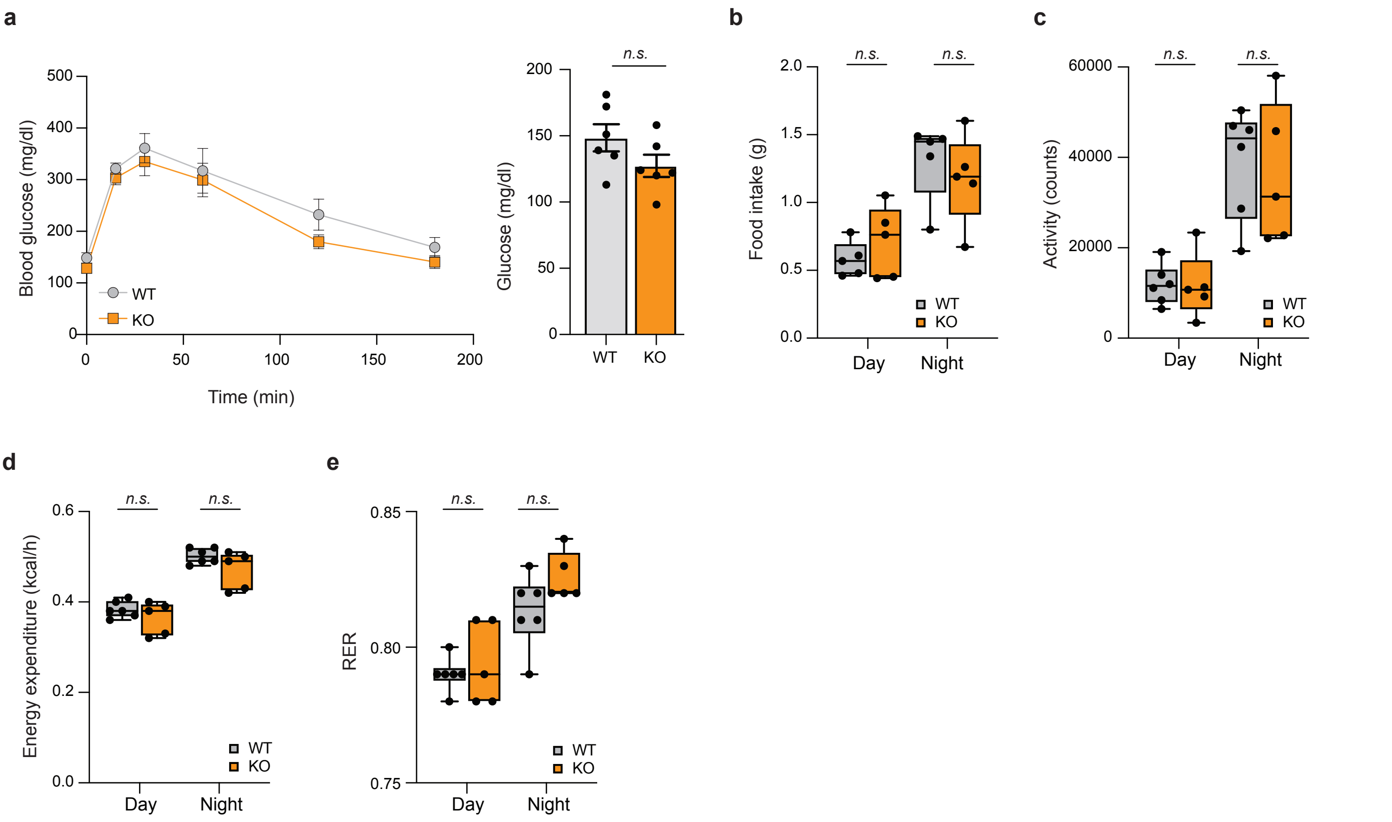

Extended Data Fig. 2: Metabolic phenotyping of *PARK7* knockout mice on HFHS diet. Measurements of (**a**) glucose tolerance and basal 6h-fasted glucose levels, (**b**) daily food intake, (**c**) locomotor activity, (**d**) energy expenditure, and (**e**) respiratory exchange ratio (*RER*) from WT and *Park7* KO mice fed a HFHS diet. Data refer to mean ± SEM, or whiskers showing min to max and boxes showing lower and higher quartiles and middle line as median (n = 5-6 per group) and were analyzed using unpaired t-test or two-way ANOVA with Tukey's multiple comparisons test; *n.s.* not significant.

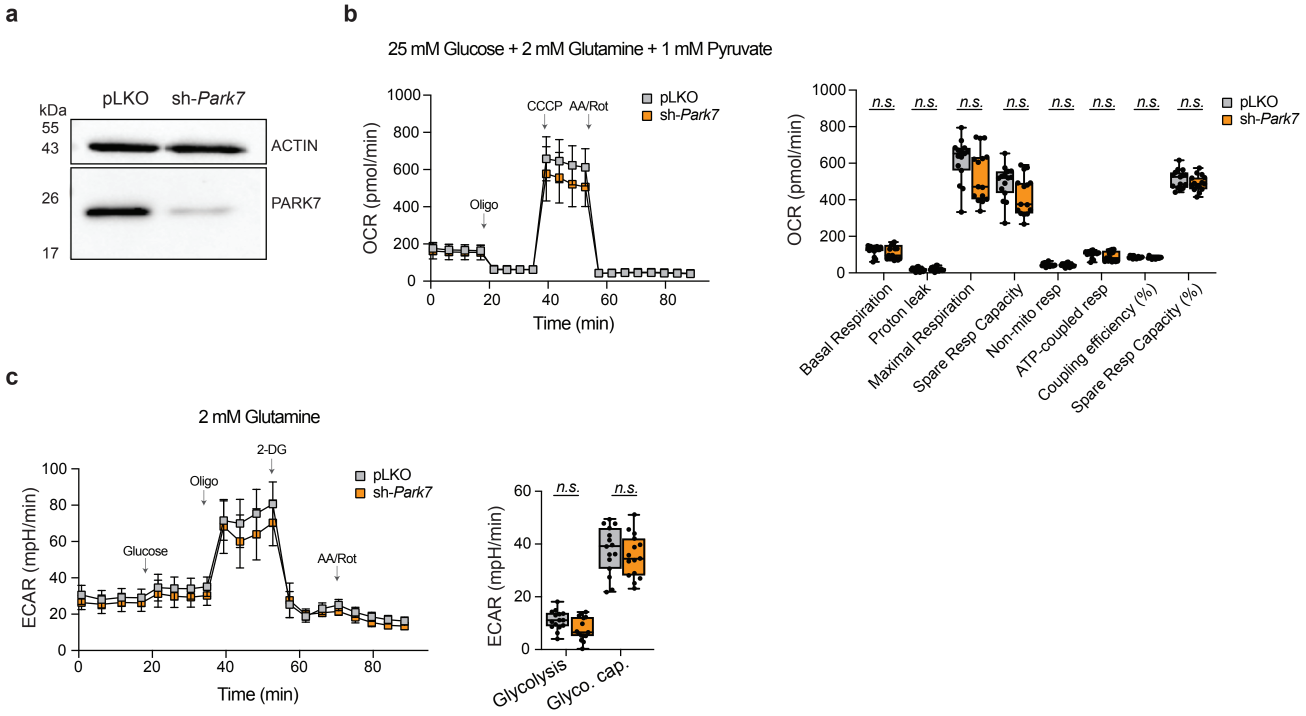

Extended Data Fig. 3: Mitochondrial bioenergetics and glycolytic metabolism are preserved in C2C12 myotubes upon *Park7* loss-of-function. (**a**) PARK7 abundance in whole cell lysates from C2C12 myotubes infected with single hairpin (sh)-RNAs targeting either empty vector (pLKO) or *Park7* (sh-*Park7*). Actin is used as a loading control. (**b**) oxygen consumption rate (OCR) average traces (*left*) and quantification (*right*) in control (pLKO) and sh-*Park7* C2C12 myotubes (n= 15) in presence of 25 mM glucose/2 mM Glutamine/1 mM Pyruvate after injection of oligomycin (Oligo, 1.5 µM), CCCP (1.5 µM), and antimycin A/rotenone (AA/Rot, 4 µM/2 µM). (**c**) Average traces (*left*) and quantification (*right*) of extracellular acidification rate (ECAR) in control (pLKO) and sh-*Park7* C2C12 myotubes (n= 15) in presence of 2 mM Glutamine after addition of glucose (10 mM), oligomycin (Oligo, 1.5 µM), 2-deoxyglucose (2-DG, 100 mM) and antimycin A/rotenone (AA/Rot, 4 µM/2 µM). *Glyco. cap.*, glycolytic capacity. Data refer to mean ± SEM or whiskers showing min to max and boxes showing lower and higher quartiles and middle line as median. Data were analyzed using multiple unpaired t-test with Holm-Sídák multiple comparisons test; *n.s.*, not significant.

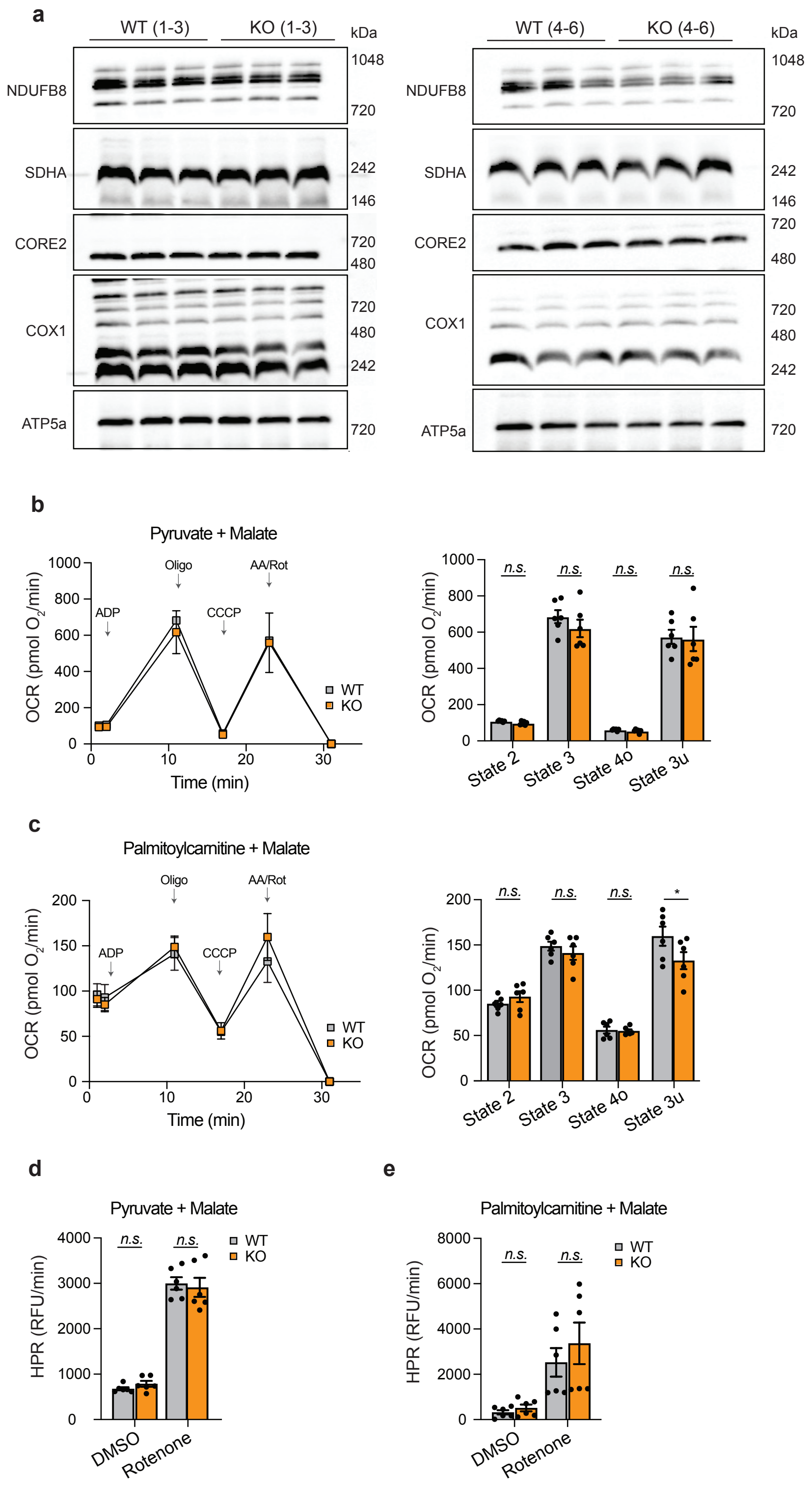

Extended Data Fig. 4: Mitochondrial bioenergetics are also preserved in gastrocnemius muscle of *Park7* KO mice. (**a**) BN-PAGE analysis of respiratory chain complex I, II, III, IV and V level and assembly in mitochondria isolated from gastrocnemius muscles of WT and *Park7* KO mice (n= 6). (**b**) Average traces (*left*) and quantification (*right*) of oxygen consumption rate (OCR) by crude mitochondria isolated from gastrocnemius muscles of WT and *Park7* KO mice (n= 6) in presence of substrates specific for respiratory chain complex I (10 mM pyruvate/2 mM malate) or (**c**) beta-oxidation (25 uM palmitoylcarnitine/2 mM malate). Substrate-dependent respiration (State 2), maximal mitochondrial respiration (State 3), resting respiration (State 4o), and uncoupled respiration (State 3u) are measured in response to 4 mM adenosine diphosphate (ADP), 1.5 uM oligomycin (Oligo), and 15 uM CCCP, respectively. Nonmitochondrial respiration is measured upon inhibition of oxidative phosphorylation by 4 µM antimycin A/2 µM rotenone (AA/Rot). (**d,e**) Horseradish peroxidase (HPR)-based quantification of reactive oxygen species produced by mitochondria isolated from gastrocnemius muscles of WT and *Park7* KO mice (n = 6) in response to either DMSO (vehicle) or 2 µM rotenone. *RFU*, relative fluorescence intensity; Data refer to mean ± SEM and were analyzed using 2way ANOVA with Sídák's multiple comparisons test (**b,c**) or two-way ANOVA with Tukey's multiple comparisons test (**d,e**); $^{*}p \leq 0.05$; *n.s.*, not significant.

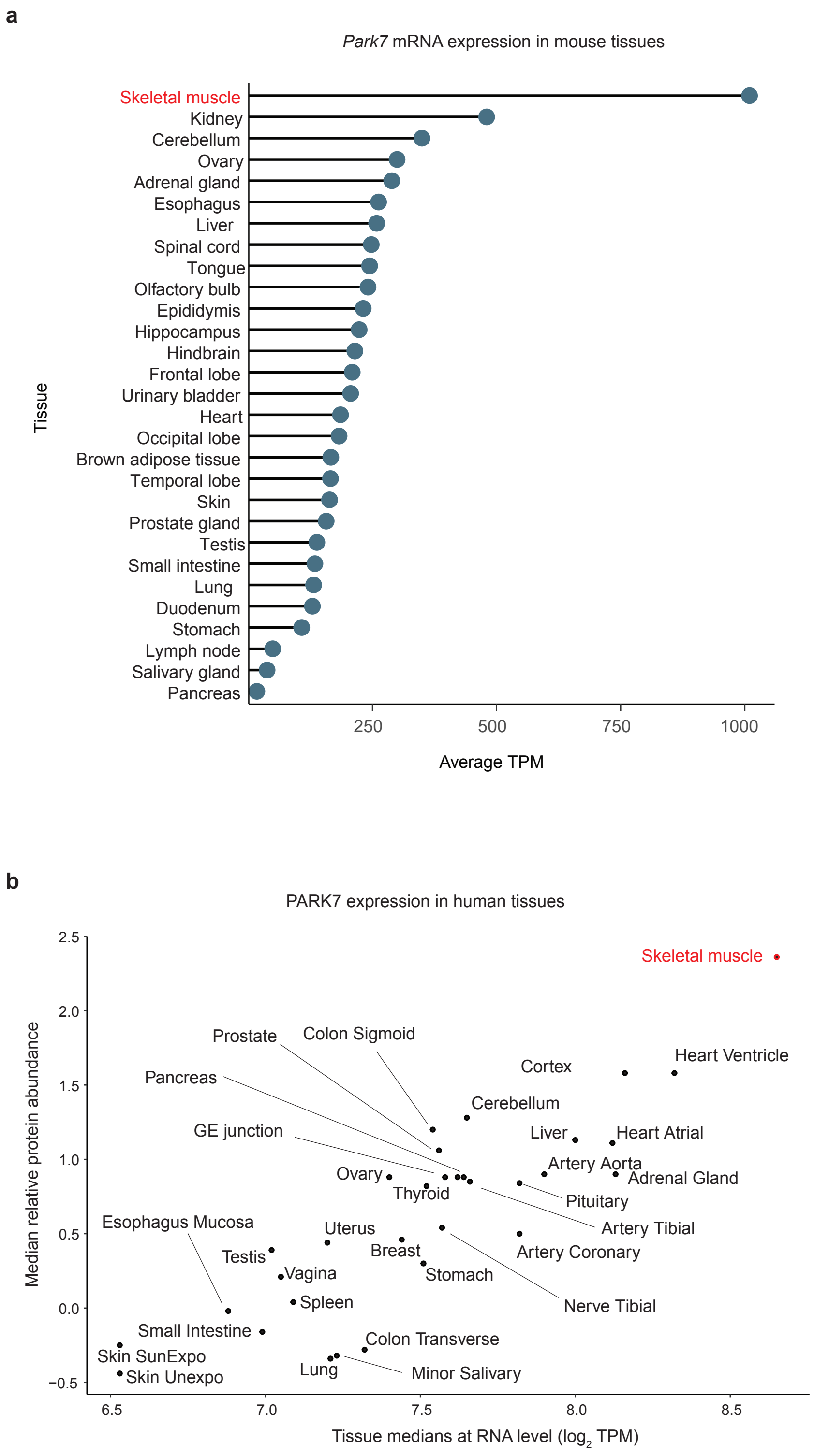

Extended Data Fig. 5: *Park7* is highly expressed in mouse skeletal muscles. (a) Median normalized expression of *Park7* mRNA (Transcripts Per Million, TPM) in mouse tissues (data from http://www.proteomicsdb.org). (**b**) Relative PARK7 protein abundance and *PARK7* mRNA expression (log_2 normalized; Transcripts Per Million, TPM) in human tissues[25].

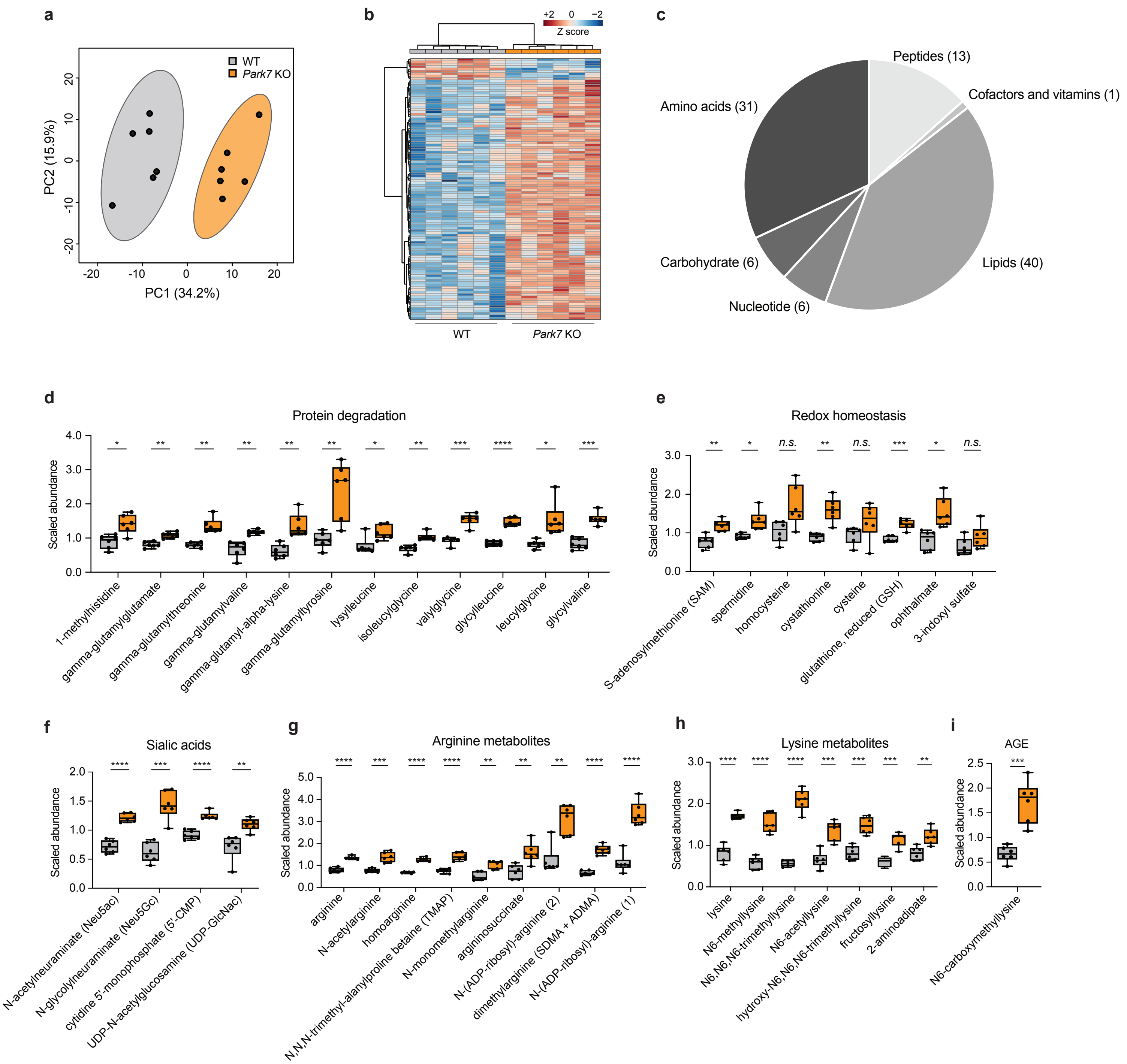

Extended Data Fig. 6: PARK7-deficiency results in profound alterations of protein and amino acid metabolism, redox homeostasis, and advanced glycation end products. (**a**) Principal component analysis of untargeted metabolomics data generated by reversed phase-ultrahigh-performance liquid chromatography-tandem mass spectrometry (RP-UHPLC-MS/MS) of gastrocnemius muscles from WT and KO mice on

HFHS diet. (**b**) Heatmap of 97 metabolites selected by random forest and shown for individual samples. (**c**) Distribution of selected metabolites in metabolic classes. Metabolite differences largely reflected protein degradation (**d**), altered redox environment (**e**), ROS-scavenging sialic acids (**f**), arginine (**g**) and lysine metabolites (**h**), and glycative stress (**i**). Whiskers show min to max, boxes represent lower and higher quartiles, and middle line is median. Data were analyzed using multiple unpaired t-test with Holm-Sídák multiple comparisons test or unpaired t-test; ****$p \leq 0.0001$, ***$p \leq 0.001$, **$p \leq 0.01$, *$p \leq 0.05$, *n.s.* not significant.

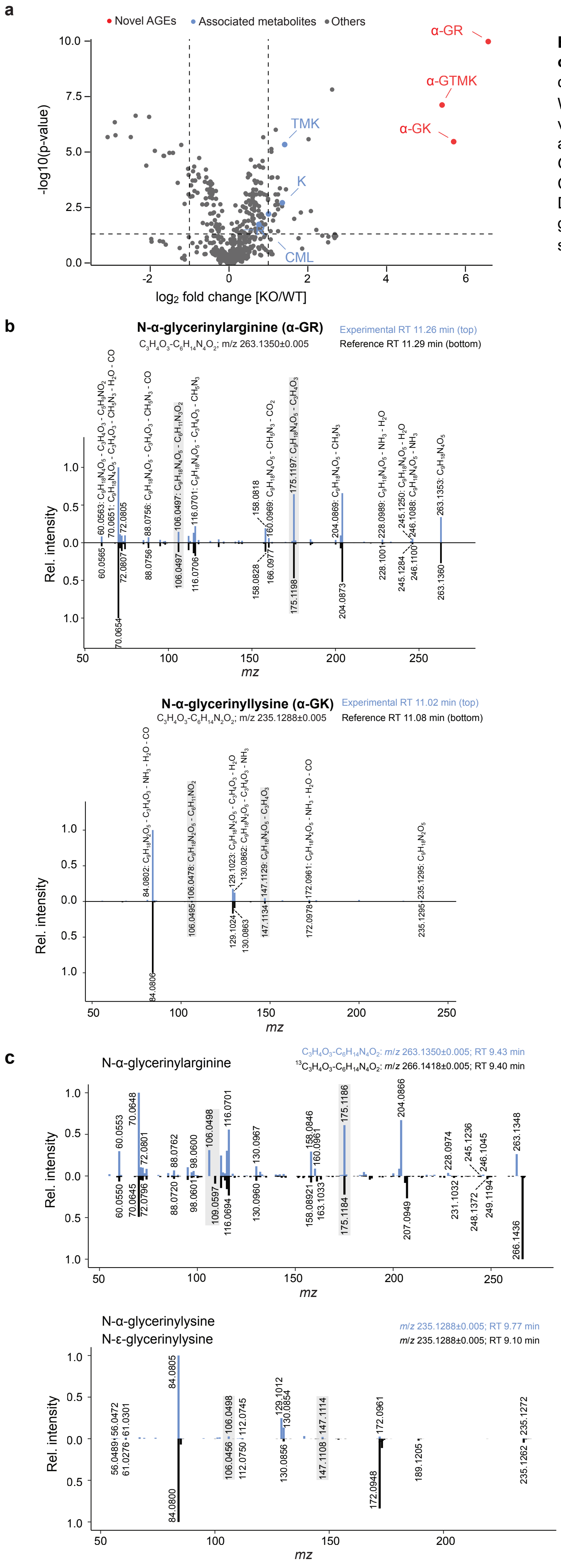

Extended Data Fig. 7: Structural confirmation of the new glycerinyl-AGEs. (**a**) Volcano plot comparing metabolomes of gastrocnemius from WT and KO mice under chow diet. (**b**) Structural verification with commercially synthesized authentic standards N-α-glycerinylarginine (α-GR) and N-α-glycerinyllysine (α-GK). (**c**) Confirmation of structures by comparison with D-glucose-$^{13}C_6$ isotopically labeled N-α-glycerinylarginine (α-GR) and commercially synthesized N-ε-glycerinyllysine (ε-GK).

de Mello et al._Extended Data Figure 7
